# Novel Tubular Biomarkers Predict Renal Progression in Type 2 Diabetes Mellitus: A Prospective Cohort Study

**DOI:** 10.1155/2016/3102962

**Published:** 2016-09-08

**Authors:** Bancha Satirapoj, Kasemsan Aramsaowapak, Theerasak Tangwonglert, Ouppatham Supasyndh

**Affiliations:** Division of Nephrology, Department of Medicine, Phramongkutklao Hospital and College of Medicine, Bangkok, Thailand

## Abstract

*Background.* Tubulointerstitial injury is both a key feature of diabetic nephropathy and an important predictor of renal dysfunction. Novel tubular biomarkers related to renal injury in diabetic nephropathy could improve risk stratification and prediction.* Methods.* A total of 303 type 2 diabetic patients were followed up. The baseline urine values of cystatin-C to creatinine ratio (UCCR), angiotensinogen to creatinine ratio (UANG), NGAL to creatinine ratio (UNGAL), and KIM-1 to creatinine ratio (UKIM-1) were measured. The primary outcome was a decline in estimated GFR of ≥25% yearly from baseline.* Results.* Urine tubular biomarkers of UCCR, UANG, UNGAL, and UKIM-1 were significantly higher according to the degree of albuminuria and all were significantly higher among patients with rapid decline in estimated GFR of ≥25% yearly from baseline. All biomarkers predicted primary outcomes with ROC for UCCR of 0.72; 95% CI 0.64–0.79, for UANG of 0.71; 95% CI 0.63–0.79, for UNGAL of 0.64; 95% CI 0.56–0.72, and for UKIM-1 of 0.71; 95% CI 0.63–0.79. Using multivariate Cox regression analysis, the number of patients with rapid renal progression was higher among those in the upper quartiles of all biomarkers than in those in the lower quartiles.* Conclusions*. Type 2 diabetic patients with high levels of urine tubular biomarkers had a more rapid decline in renal function.

## 1. Introduction

Diabetic nephropathy (DN) is an important health problem in the worldwide adult population, and approximately 20–40% of DN patients inevitably progress to end stage renal disease (ESRD) [1]. Albuminuria is an important marker to diagnosis and predicts the progression of DN [2, 3], but some studies have shown that only 30–45% of diabetic patients with microalbuminuria have progressed to macroalbuminuria in 10-year follow-up; 30% of them regressed to normoalbuminuria, and 30–40% remained at microalbuminuria level [4]. Furthermore, 20% of type 2 diabetic (T2DM) patients had their glomerular filtration rate (GFR) decline before detecting albuminuria. Therefore, identifying new biologic markers involved in DN progression is needed.

All the renal cellular elements including the glomerular endothelium, mesangial cells, podocytes, and tubular epithelium are affected in setting of chronic hyperglycemia. Furthermore, renal tubular and interstitial compartments have been increasingly reported to play an integral role in the pathogenesis of DN and they correlate well with progressive renal function decline and represent a final common pathway of chronic kidney disease (CKD) [5]. In T2DM, novel tubular biomarkers that relate to renal tubular injury could improve risk stratification and prediction.

Neutrophil gelatinase associated lipocalin (NGAL) is expressed in the renal tubular epithelium, and a rise in urinary concentrations may provide a potential biomarker of a chronically injured kidney [6]. Kidney Injury Molecule-1 (KIM-1) is a type 1 membrane protein expressed on the apical membrane of proximal tubule cells. Its ectodomain is cleaved and released in the lumen of the tubule and finally appears in urine, which is stable [7]. Cystatin-C is produced from nucleated cells in the body and easily filtered by the glomeruli and is reabsorbed and catabolized by the proximal tubule. Previous study showed that the urine Cystatin-C (uCys-C) level represented an indicator of renal tubular dysfunction [8]. Finally, changes in angiotensinogen could influence RAS activity and increases in intrarenal RAS components, in parallel with the severity of fibrotic renal damage, have been demonstrated in chronic progressive nephropathy [9, 10]. Thus, all tubular biomarkers serve as a promising biomarker for tubule damage.

Starting from renal tubular injury playing a role in the pathogenesis of DN and progressive decline of renal function, we hypothesized that baseline urinary tubular injury biomarkers are an important determinant of lower GFR in a wider cohort of T2DM patients. However, inconsistent results have been produced with regard to predictors of all biomarkers in type 2DM with nephropathy.

## 2. Materials and Methods

### 2.1. Study Populations

Age, sex, and diabetes duration matched subjects with various stages of normoalbuminuria (urine albumin creatinine ratio (UACR) < 30 mg albumin/g creatinine, *N* = 94), microalbuminuria (UACR 30–300 mg albumin/g creatinine, *N* = 95), and macroalbuminuria T2DM (UACR > 300 mg albumin/g creatinine and/or persistent proteinuria, *N* = 114) were recruited in February 2014 and March 2015 and followed up for a least 12 months at the outpatient clinic, Department of Internal Medicine, Phramongkutklao Hospital. The study was approved by the Ethics Committee of the Institute Review Board at the Royal Thai Army Medical Department and all patients gave written informed consent. Inclusion criteria included age ≥18 years and T2DM. Exclusion criteria included acute kidney injury (AKI) episode, pregnancy, unspecified type of DM, and patient life expectancy <1 year. All patient histories were carefully recorded by interview and confirmed by checking patient records and recording drug prescriptions. Clinical examination, including assessment of body mass index (BMI), systolic and diastolic blood pressure (BP), fasting plasma glucose, and other basic laboratory data, was conducted. BP was measured three times, and the average value was used to analyze data.

### 2.2. Laboratory Measurements

Blood samples were taken in the morning before any food intake. Common biochemical parameters including urea, creatinine, hemoglobin A1C, serum lipids and electrolytes, albumin, hemoglobin, and proteinuria were measured at baseline in all patients, according to standard methods in a routine clinical laboratory. Estimated GFR was assessed using the Chronic Kidney Disease Epidemiology Collaboration (CKD-EPI) equation [11]. Urine albumin was measured on a nephelometric analyzer and urine creatinine was measured on a multiple analyzer (Modular P Chemistry Analyzer; Roche Diagnostics). Urine albumin and creatinine for urine samples collected from participants and albuminuria were reported as albumin creatinine ratio (UACR).

### 2.3. Urine Tubular Biomarkers

Urine tubular biomarkers were collected at baseline. Thirty milliliters of fresh urine was centrifuged at 4,000 rpm for 10 minutes and then stored at −80°C until assayed. All tubular biomarkers were tested by a commercially available sandwich ELISA kit. All specimens were diluted often to obtain concentration at the optimal density according to the ELISA kit instruction. Coefficients of variation for urine tubular biomarkers assays were <10%, for intra-assay and interassay variation. The enzymatic reactions were quantified in an automatic microplate photometer. All measurements were made in triplicate and blinded manner. Urine NGAL (R&D Systems Inc., USA and Canada) and KIM-1 (R&D Systems Inc., USA and Canada) levels were expressed as nanograms per gram of creatinine (UNGAL and UKIM-1). Cystatin-C (R&D Systems China Co., Ltd) levels were expressed as micrograms per gram of creatinine (UCCR). Urine angiotensinogen (R&D Systems China Co., Ltd) by solid phase ELISA technique was expressed as nanograms per gram of creatinine (UANG).

### 2.4. Renal Outcome

After the baseline assessments, patients were followed up prospectively until the end of the observation period. The latter was defined by the combined outcomes of percentage changes of GFR decline from baseline and rapid renal progression was defined by decreased estimated GFR ≥25% from baseline in one year. Patients were personally contacted in case they missed any appointment and at the end of the study, to avoid eventual loss during follow-up.

### 2.5. Statistical Analyses

Data were presented as mean ± SD, median, or percentage frequency, as appropriate. Differences between groups were established by unpaired *t*-test or chi square test. Receiver operating characteristic (ROC) analysis was used to calculate the area under the curve (AUC) for UNGAL, UKIM-1, UCCR, UANG, and UACR, and to find the best cut-off values of other tubular biomarkers to identify the progression to renal endpoint. Kaplan-Meier curves were generated to assess renal survival in subjects with each cut-off point values of tubular biomarkers above and below the optimal ROC-derived cut-off levels. For examination of associations with decline GFR and quartiles of urinary biomarkers, we first examined the unadjusted relationships and then adjusted the models for BMI, systolic BP, anemia, RAAS blocker, serum creatinine, and UACR by multivariate Cox proportional hazard regression analysis. All results were considered significant when *P* was <0.05.

## 3. Results

### 3.1. Patients Baseline Characteristics

A total of 303 subjects (96%) at the second visit of the study were recruited for analysis. The baseline characteristics of the study cohort are summarized in Table 1. Mean age of patients was 66.4 ± 11.4 years, and more than half were male (55.4%). Onset of T2DM was 12.2 ± 9.2 years, all patients had follow-up time of 12.3 ± 4 months. Regarding clinical and laboratory data, mean estimated GFR was 50 ± 29.7 mL/min/1.73 m^2^, mean UACR was 887 ± 210 mg/gCr, and HbA1c was 7.3 ± 1.5%.

### 3.2. Progression Endpoint during the Follow-Up Period

During the observational period (median follow-up of 12.3 ± 4 months), 41 patients (13.5%) had GFR decline ≥25% yearly from baseline as rapid renal progression. The patients with rapid renal progression had a higher prevalence of anemia; RAAS blocker usage increased systolic BP and decreased baseline estimated GFR and baseline UACR (Table 1).

**Figure 1:**
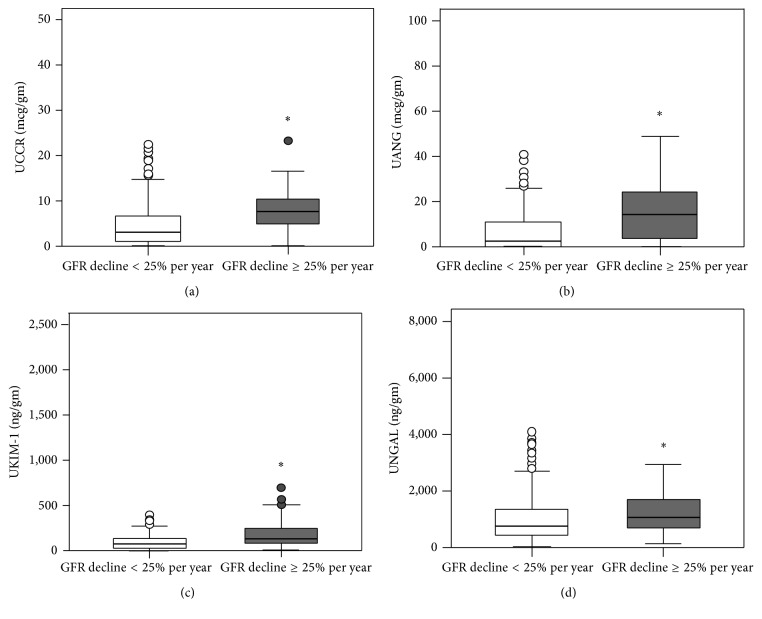
Urinary levels of tubular biomarkers in the rapid and nonrapid GFR decline groups. (a) Cystatin-C, (b) Angiotensinogen, (c) KIM-1, and (d) NGAL adjusted by urinary creatinine in T2DM patients classified in two groups according to GFR decline: rapid renal progression, nonrapid renal progression. Results are presented as median. UANG: urine angiotensinogen creatinine ratio; UCCR: urine cystatin-C creatinine ratio; UKIM-1: Urine Kidney Injury Molecule-1 creatinine ratio; UNGAL: Urine Neutrophil Gelatinase Associated Lipocalin creatinine ratio. ^*∗*^ *P* < 0.01 versus GFR decline < 25% per year.

Urine tubular biomarker levels of UCCR, UANG, UKIM-1, and UNGAL were significantly higher in the rapid renal progression group when compared with nonrapid renal progression group, represented by median (interquartile range at 25%–75%) value of UCCR 3.01 [1.04–17.15]* versus* 7.52 [4.74–16.49] mcg/gm (*P* < 0.001), UANG 2.41 [0.31–10.73]* versus* 14.26 [3.29–24.48] mcg/gm (*P* < 0.001), UKIM-1 67.5 [32.1–132.2]* versus* 133.1 [81.9–255.6] ng/gm (*P* < 0.001), and UNGAL 751.3 [413.9–1350.5]* versus* 1058.3 [702.1–1693.3] ng/gm (*P* = 0.004) as shown in Figure 1. Macroalbuminuric and microalbuminuric T2DM patients had higher levels of all urine tubular biomarkers as compared to normoalbuminuric patients. However, in subgroup of albuminuria, there were no significant differences in the urine tubular biomarkers between rapid and nonrapid renal progression groups. The relative small sample size and few reported end points were detected in each subgroup (Table 2).

### 3.3. Performance of Urine Tubular Biomarkers with Rapid CKD Progression

**Figure 2:**
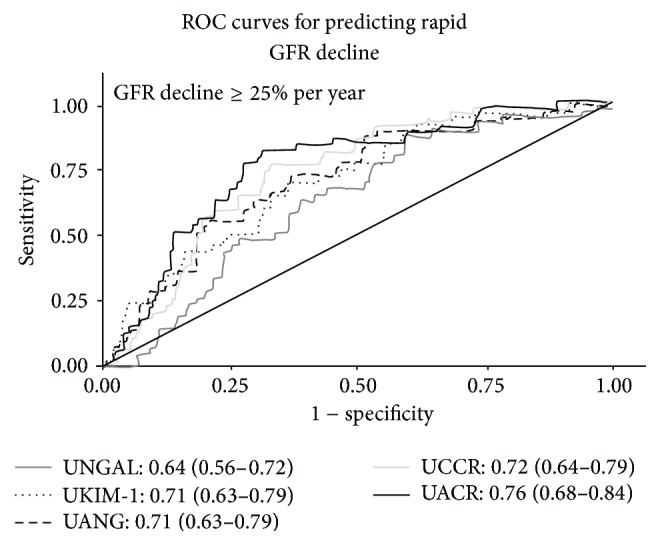
Graph ROC curves showing Area under the Curve (AUC) of each tubular biomarker to predict rapid GFR decline. UACR: urine albumin creatinine ratio; UANG: urine angiotensinogen creatinine ratio; UCCR: urine cystatin-C creatinine ratio; UKIM-1: Urine Kidney Injury Molecule-1 creatinine ratio; UNGAL: Urine Neutrophil Gelatinase Associated Lipocalin creatinine ratio.

ROC analysis showed an AUC for UCCR, UANG, UKIM-1, UNGAL, and UACR of 0.72 (95% CI: 0.64 to 0.79), 0.71 (95% CI: 0.63 to 0.79), 0.71 (95% CI: 0.63 to 0.79), 0.64 (95% CI: 0.56–0.72), and 0.76 (95% CI: 0.68–0.84), respectively, as shown in Figure 2. For UCCR, the best cut-off level was 3.43 mcg/g (sensitivity 80.5%, specificity 56.9%), for UANG was 4.52 mcg/g (sensitivity 73.2%, specificity 63.4%), for UKIM-1 was 95 ng/g (sensitivity 70.7%, specificity 63.4%), and for UNGAL was 772 ng/g (sensitivity 68.3%, specificity 51.1%). All tubular biomarkers demonstrated intermediate performance to predict rapid renal progression in T2DM patients.

**Figure 3:**
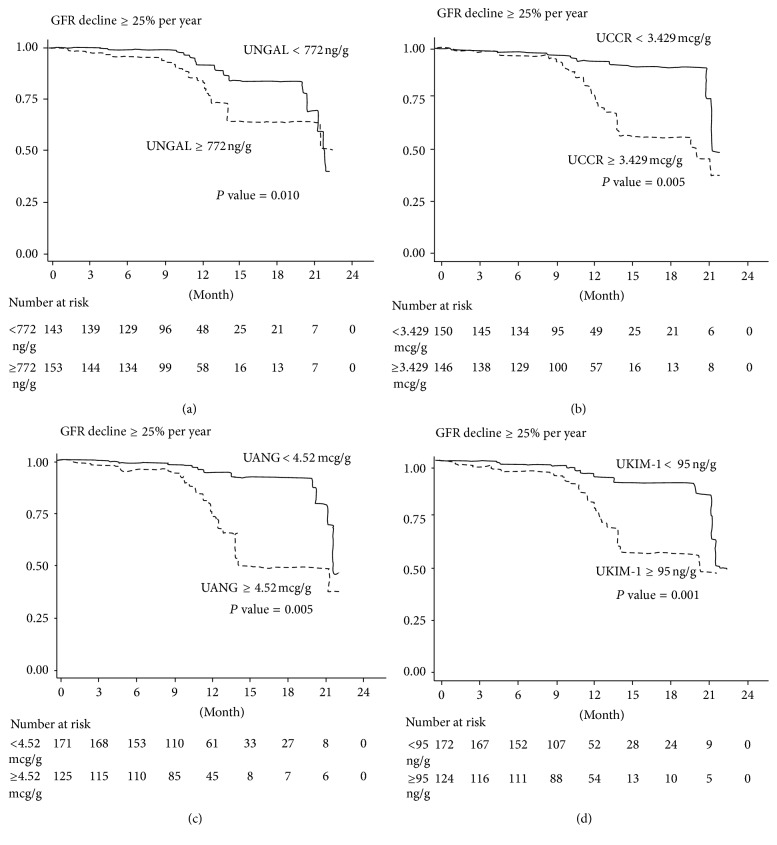
Kaplan-Meier survival curves of renal endpoint in patients with UNGAL, UCCR, UANG, and UKIM-1 levels above and below the optimal receiver operating characteristics cut-off level of each tubular biomarker. (a) Patients with UNGAL ≥772 ng/g (*P* = 0.01, log-rank test), (b) UCCR ≥ 3.429 ng/g (*P* = 0.005, log-rank test), (c) UANG ≥4.52 mcg/g (*P* = 0.005, log-rank test), and (d) UKIM-1 ≥95 ng/g (*P* = 0.001, log-rank test) showed a significantly faster progression to endpoint. UANG: urine angiotensinogen creatinine ratio; UCCR: urine cystatin-C creatinine ratio; UKIM-1: Urine Kidney Injury Molecule-1 creatinine ratio; UNGAL: Urine Neutrophil Gelatinase Associated Lipocalin creatinine ratio.

Kaplan-Meier survival curves in patients with UCCR, UANG, UKIM-1, and UNGAL levels above and below the optimal cut-off are presented in Figure 3. Subjects with UCCR, UANG, UKIM-1, and UNGAL values above 3.43 mcg/g, 4.52 mcg/g, 95 ng/g, and 772 ng/g, respectively, experienced a significantly faster evolution to endpoint (*P* < 0.05) with a mean follow-up time to progression of 12 months.

### 3.4. Cox Regression Analysis and Progression of CKD

To identify putative risk factors associated with the progression of CKD, we performed a Cox regression analysis in the model for all variables that differed at baseline in patients who reached the endpoint during the whole follow-up period (anemia, systolic BP, RAAS blocker usage, baseline serum creatinine, and UACR). Univariate analysis showed significantly increased risk for rapid CKD progression in the group of upper quartiles of all urine tubular biomarkers when compared with lower quartiles (Table 3). Multiple Cox regression analysis after adjusting potential risks for CKD progression revealed that all urine tubular biomarkers including UCCR, UANG, UKIM-1, and UNGAL were significantly associated with rapid CKD progression at the end of the study.

## 4. Discussion

Findings from the present study clearly indicate that all novel tubular biomarkers, that is, UCCR, UANG, UKIM-1, and UNGAL, represent novel risk markers of DN progression. All novel urinary tubular biomarkers showed a most impressive predictive power in such a contest even after adjusting for conventional risk factors (baseline BMI, systolic BP, anemia, RAAS blocker used, serum creatinine, and UACR). This suggests that all novel tubular biomarkers would not be simple surrogate indexes of baseline estimated GFR, but markers on their own, predicting DN progression beyond the information provided by serum creatinine and other conventional risk factors.

In recent years, however, different studies have underlined the crucial role played by the renal tubule in the genesis of progressive acute and chronic kidney disease and its evolution to terminal stage [12]. Importantly, in diabetic with renal pathology, renal function correlated better with the degree of tubule-interstitial lesions than with that of the glomerular lesions [13]. New biomarkers of the processes that induce these tubulointerstitial changes may ultimately prove to be better predictors of disease progression and long-term prognosis than our current markers [14].

Cystatin-C, angiotensinogen, NGAL, and KIM-1 are known markers of acute kidney injury. All of these markers are upregulated in renal tubules after renal injury [15–18]. Cystatin-C, angiotensinogen, and KIM-1 markers located in the proximal tubules following renal injury, whereas NGAL was defined in the distal tubules. Recent literature suggests that all of these may be a marker to detect progression of CKD. There are limited studies on the association between tubular biomarkers and CKD progression.

Prior cross-sectional studies examining the increased urine tubular biomarkers include cystatin, KIM-1, NGAL, and angiotensinogen in early CKD and early DN [19–22]. Similarly, in one-year observational follow-up study in 74 T2DM patients with nephropathy, high urine NGAL levels at baseline correlated with declined levels of estimated GFR and increased serum creatinine [23]. One study in T2DM demonstrated that KIM-1 predicted the decline of GFR in unadjusted analysis [24] and two studies in T1DM also showed that KIM-1 levels were significantly higher in the patients who progressed from nonmacroalbuminuria to CKD stage 3 [25] and macroalbuminuria to late stage of CKD [26]. In addition, urine cystatin-C as reported from a prospective study predicted the CKD progression in the largest study reported to date, with 237 patients with T2DM [27]. Therefore, our study measured all of promising urinary tubular biomarkers at the same time in T2DM patients. Our results are consistent with those in additional studies, and we provide more validation for these biomarkers associated with the progressive course of the disease.

In this study, we found that T2DM with rapid renal progression had significantly increased levels of urinary biomarkers when compared with the nonrapid renal progression group. From ROC curves (AUC 0.64–0.72) each tubular biomarker presented an intermediate accuracy performance in predicting rapid renal progression and all tubular biomarkers had an AUC less than the AUC of UACR (0.76) possibly affecting the level of proteinuria in our population. Cox proportional hazard models, representing all novel tubular biomarkers, were independent predictors of CKD progression in T2DM. Our study integrated novel tubular biomarkers at the same time and compared the performance of each tubular biomarker with the standard urinary biomarker, UACR. Two studies in early CKD and short-term (less than five years) duration of T2DM showed that only increased urine NGAL, but not urine KIM-1, was significantly associated with GFR decline [28, 29]. As opposed to previous studies in participants with CKD [28] and early stage of DN [29], all tubular biomarkers levels in our study were significantly correlated with GFR decline and albuminuria. A significant rise in urinary tubular levels may indicate severe tubular cells damage in setting of established nephropathy in T2DM population.

Study limitations included being a single center study and having a short follow-up period of 12 months. A long-term follow-up and multicenter study is recommended. However, in our study, urine biomarkers were measured in a cohort of both early and advanced DN patients who might have a more rapid decline in renal function in limited time of follow-up. Our primary endpoint was also reached by fifteen percent of the patients, and the statistical model was powerful enough to establish independent relationships between biomarkers and GFR decline in T2DM. Our study did not collect data of main renal outcomes for demonstrating the doubling of serum creatinine and initiating long-term dialysis. The relatively small sample size of our study limits the precision and power to detect associations of moderate strength. Follow-up was based on a single serum creatinine and GFR calculation for detecting a decrease in GFR of >25% from baseline; it may have some potential bias due to imprecision in estimated GFR. Finally, our data suggest that all the biomarkers measured in the urine are derived mostly or entirely from a renal tubular source. However, the data do not definitively exclude the possibility that some fraction of the excreted biomarkers especially plasma KIM-1 may be derived from glomerular filtration. Animal experimental study addressed that plasma KIM-1 was highly increased in diabetic animals compared to nondiabetic animals [30]. Another study also indicated that plasma KIM-1 level predicted rate of GFR decline and incidence of ESRD in a cohort of T1DM patients with advanced nephropathy [31].

In conclusion, T2DM patients with high levels of urine tubular biomarkers (urine cystatin-C, angiotensinogen, KIM-1, and NGAL) presented more rapid decline in renal function. All urine tubular biomarkers were independent predictors of rapid renal progression among T2DM patients. For future study, in early stage T2DM in the normo- or microalbuminuria group, novel tubular biomarkers may possibly have a role in early detection compared to the conventional marker to predict CKD progression.

## Figures and Tables

**Table 1:** Baseline characteristics and laboratory data.

Parameters	All patients	GFR decline < 25% per year	GFR decline ≥ 25% per year
	*N* = 303	(*N* = 262)	(*N* = 41)
Age (year)	66.4 ± 11.4	67 ± 11.3	64.5 ± 10.3
Male (%)	55.4	53.4	68.3
Duration of DM (years)	12.2 ± 9.2	12.2 ± 9.3	12.5 ± 8.9
Time F/U (month)	12.3 ± 4	12.1 ± 4.5	12.5 ± 5.0
Comorbid disease			
CKD staging			
CKD I	38 (12.5%)	35 (13.4%)	3 (7.3%)^*∗*^
CKD II	71 (23.4%)	68 (26.0%)	3 (7.3%)^*∗*^
CKD III	102 (33.7%)	89 (34.0%)	13 (31.7%)^*∗*^
CKD IV	55 (18.2%)	43 (16.4%)	12 (29.3%)^*∗*^
CKD V	37 (12.2%)	27 (10.3%)	10 (24.4%)^*∗*^
Hypertension (%)	95.6	95.7	95
Dyslipidemia (%)	91.2	92.6	85
Cardiovascular disease (%)	19.5	17.2	26.8
Anemia (%)	53.9	50.1	78.0^*∗*^
Medications			
RAAS blocker (%)	58.1	60.6	42.5^*∗*^
Insulin (%)	27.7	25.5	40.0
ASA (%)	61	61.8	60.0
Clinical parameter			
SBP (mmHg)	139.8 ± 19.6	138.7 ± 18.6	147.6 ± 24.1^*∗*^
DBP (mmHg)	76.2 ± 12.3	75.6 ± 11.8	79.7 ± 15.5
BMI (kg/m^2^)	26.89 ± 4.2	26.71 ± 4.56	28.05 ± 5.41
Laboratory parameter			
GFR (mL/min/1.73 m^2^)	49.88 ± 29.71	51.98 ± 29.36	33.40 ± 25.46^*∗*^
UACR (mg/g)	887.0 ± 210.8	742.6 ± 210.3	1885.4 ± 214^*∗*^
FPG (mg/dL)	143.7 ± 62.3	141.6 ± 61.7	156.9 ± 69.0
HbA1c (%)	7.3 ± 1.5	7.2 ± 1.5	7.5 ± 1.5
Hemoglobin (g/dL)	12.1 ± 2.6	12.2 ± 2.1	11.7 ± 3.8
Phosphate (mg/dL)	3.51 ± 0.8	3.53 ± 0.8	3.43 ± 0.9
Intact-PTH (pg/mL)	152.9 ± 149	127.7 ± 111.2	239.4 ± 231.2

^*∗*^ *P* < 0.05 versus group with GFR decline < 25% per year.

Note: values for categorical variables are given as number (percentage); values for continuous variables are given as mean ± standard deviation or median [interquartile range].

ASA: aspirin; BMI: body mass index; DBP: diastolic blood pressure; FPG: fasting plasma glucose; GFR: glomerular filtration rate; HbA1c: hemoglobin A1 C; PTH: parathyroid hormone; RAAS: renin angiotensin aldosterone system; SBP: systolic blood pressure; UACR: urine albumin creatinine ratio.

**Table 2:** Urinary levels of tubular biomarkers with different stages of albuminuria in the rapid and nonrapid GFR decline groups.

Urine biomarkers	*N*	GFR decline < 25% per year	*N*	GFR decline ≥ 25% per year	*P* value
		Median (IQR)		Median (IQR)	
UCCR (mcg/gm)					
(i) All patients (*N* = 303)	262	3.01 (1.04, 6.46)	41	7.52 (4.74, 10.27)	<0.001^*∗*^
(ii) Normoalbuminuria (*N* = 94)	90	1.50 (0.21, 3.25)	4	3.93 (1.87, 7.67)	0.116
(iii) Microalbuminuria (*N* = 95)	91	2.43 (0.95, 3.73)	4	2.36 (0.77, 4.36)	0.950
(iv) Macroalbuminuria (*N* = 114)	81	7.60 (4.23, 12.71)	33	8.11 (6.07, 11.61)	0.696
UANG (mcg/gm)					
(i) All patients (*N* = 303)	262	2.41 (0.31, 10.73)	41	14.26 (3.29, 24.48)	<0.001^*∗*^
(ii) Normoalbuminuria (*N* = 94)	90	0.49 (0.11, 1.71)	4	1.77 (0.67, 5.35)	0.277
(iii) Microalbuminuria (*N* = 95)	91	1.83 (0.35, 5.82)	4	3.13 (0.79, 5.48)	0.879
(iv) Macroalbuminuria (*N* = 114)	81	13.96 (5.32, 23.21)	33	14.74 (9.26, 27.88)	0.585
UKIM-1 (ng/gm)					
(i) All patients (*N* = 303)	262	67.5 (32.1, 132.2)	41	133.1 (81.9, 255.6)	<0.001^*∗*^
(ii) Normoalbuminuria (*N* = 94)	90	37.4 (16.4, 65.9)	4	55.9 (53.2, 116.7)	0.073
(iii) Microalbuminuria (*N* = 95)	91	67.0 (32.1, 121.8)	4	53.8 (28.1, 79.2)	0.457
(iv) Macroalbuminuria (*N* = 114)	81	129.2 (88.3, 209.5)	33	178.9 (104.9, 371.8)	0.077
UNGAL (ng/gm)					
(i) All patients (*N* = 303)	262	751.3 (413.9, 1350.5)	41	1058.3 (702.1, 1693.3)	0.004^*∗*^
(ii) Normoalbuminuria (*N* = 94)	90	495.15 (342.1, 801.8)	4	662.1 (391.7, 1064.85)	0.594
(iii) Microalbuminuria (*N* = 95)	91	654.7 (390.6, 1245.7)	4	625.3 (323.55, 1144.2)	0.701
(iv) Macroalbuminuria (*N* = 114)	81	1199.6 (787.3, 1942)	33	1347.6 (886.6, 1812.6)	0.795

^*∗*^ *P* < 0.05 versus group with GFR decline < 25% per year.

Note: values for continuous variables are represented as median [interquartile range].

UANG: urine angiotensinogen creatinine ratio; UCCR: urine cystatin-C creatinine ratio; UKIM-1: Urine Kidney Injury Molecule-1 creatinine ratio; UNGAL: Urine Neutrophil Gelatinase Associated Lipocalin creatinine ratio.

**Table 3:** Cox proportional hazards modeling of quartiles of urine biomarkers to predict rapid GFR decline.

Predictor variables	UCCR (HR [95% CI])	*P*	UANG (HR [95% CI])	*P*	UKIM-1 (HR [95% CI])	*P*	UNGAL (HR [95% CI])	*P*
Unadjusted								
First quartile	Reference		Reference		Reference		Reference	
Second quartile	2.34 (0.56–9.85)	0.246	2.83 (0.73–10.98)	0.133	2.42 (0.51–11.43)	0.265	6.44 (1.43–29.11)^*∗*^	0.015
Third quartile	3.95 (1.1–14.2)^*∗*^	0.035	3.18 (0.84–12.01)	0.088	3.68 (0.81–16.78)	0.092	7.15 (1.61–31.7)^*∗*^	0.010
Fourth quartile	7.52 (2.23–25.28)^*∗*^	0.001	8.37 (2.49–28.08)^*∗*^	0.001	6.95 (1.61–29.90)^*∗*^	0.009	8.23 (1.87–36.16)^*∗*^	0.005
Adjusted for model^*∗*^								
First quartile	Reference		Reference		Reference		Reference	
Second quartile	1.99 (0.44–9.15)	0.385	2.41 (0.59–9.80)	0.218	5.36 (0.65–43.97)	0.118	5.36 (1.15–25.03)^*∗*^	0.033
Third quartile	3.76 (1.02–13.85)^*∗*^	0.046	2.64 (0.66–10.54)	0.170	8.04 (1.01–25.08)^*∗*^	0.049	4.92 (1.05–23.08)^*∗*^	0.043
Fourth quartile	5.69 (1.47–21.97)^*∗*^	0.012	6.85 (1.76–26.67)^*∗*^	0.006	9.61 (1.23–75.06)^*∗*^	0.031	3.85 (1.76–26.67)^*∗*^	0.037

^*∗*^Adjusted model with baseline of body mass index, systolic blood pressure, anemia, RAAS blocker, serum creatinine, and urine albumin creatinine ratio.

HR: hazard ratio; CI: confidence interval; UANG: urine angiotensinogen creatinine ratio; UCCR: urine cystatin-C creatinine ratio; UKIM-1: Urine Kidney Injury Molecule-1 creatinine ratio; UNGAL: Urine Neutrophil Gelatinase Associated Lipocalin creatinine ratio.
